# Targeting the Acute Promyelocytic Leukemia-Associated Fusion Proteins PML/RARα and PLZF/RARα with Interfering Peptides

**DOI:** 10.1371/journal.pone.0048636

**Published:** 2012-11-09

**Authors:** Sabine Beez, Philipp Demmer, Elena Puccetti

**Affiliations:** 1 Institute of Molecular Biology and Tumor Research, Philipps University, Marburg, Germany; 2 Department of Hematology, Goethe-University, Frankfurt, Germany; West Virginia University School of Medicine, United States of America

## Abstract

In acute promyelocytic leukemia (APL), hematopoietic differentiation is blocked and immature blasts accumulate in the bone marrow and blood. APL is associated with chromosomal aberrations, including t(15;17) and t(11;17). For these two translocations, the retinoic acid receptor alpha (RARα) is fused to the promyelocytic leukemia (PML) gene or the promyelocytic zinc finger (PLZF) gene, respectively. Both fusion proteins lead to the formation of a high-molecular-weight complex. High-molecular-weight complexes are caused by the “coiled-coil” domain of PML or the BTB/POZ domain of PLZF. PML/RARα without the “coiled-coil” fails to block differentiation and mediates an all-*trans* retinoic acid-response. Similarly, mutations in the BTB/POZ domain disrupt the high-molecular-weight complex, abolishing the leukemic potential of PLZF/RARα. Specific interfering polypeptides were used to target the oligomerization domain of PML/RARα or PLZF/RARα. PML/RARα and PLZF/RARα were analyzed for the ability to form high-molecular-weight complexes, the protein stability and the potential to induce a leukemic phenotype in the presence of the interfering peptides. Expression of these interfering peptides resulted in a reduced replating efficiency and overcame the differentiation block induced by PML/RARα and PLZF/RARα in murine hematopoietic stem cells. This expression also destabilized the PLZF/RARα-induced high-molecular-weight complex formation and caused the degradation of the fusion protein. Targeting fusion proteins through interfering peptides is a promising approach to further elucidate the biology of leukemia.

## Introduction

Acute myeloid leukemia (AML) is characterized by an accumulation of progenitor blasts in the bone marrow. These blasts are blocked in their differentiation and are characterized by an aberrant “self-renewal” [1]. Acute promyelocytic leukemia (APL) is a well-characterized subtype of AML with the specific t(15;17) and t(11;17) translocations, which are considered indispensable for the leukemic phenotype [1].

The t(15;17) and t(11;17) translocations involve the same domain of the retinoic acid receptor alpha (RARα) fused to PML or PLZF, respectively. Ectopic expression of PML/RARα and PLZF/RARα in hematopoietic cells mimics the leukemic phenotype by inducing a differentiation block and an aberrantly activated self-renewal capability, leading to the development of leukemia in mice [2], [3]. The biological activities of these fusion proteins have yet to be completely elucidated. One proposed mechanism suggested that the inhibition of differentiation-associated transcription factors could occur by direct binding, such as VDR and PU.1, leading to their sequestration [4], [5]. Treatment with ATRA (all-*trans* retinoic acid) is able to overcome the differentiation block in PML/RARα-expressing blasts but not in PLZF/RARα-positive-blasts [6]. *In vivo* PML/RARα forms high-molecular-weight (HMW) complexes through the “coiled-coil” region of PML [7]. Furthermore, the PML “coiled-coil” region of PML/RARα is required to block the terminal differentiation in PML/RARα positive blasts [7], [8]. Treatment with ATRA disrupt the formation of HMW complexes by PML/RARα [9]. PLZF/RARα is able to form HMW complexes through the BTB/POZ domain [9]. Crystallization studies on the BTB/POZ domain showed that the POZ domain exhibits a structure that enables the formation of homodimers, which assume a quaternary structure to form a multimeric complex [10], [11].

Another common feature of both PML/RARα and PLZF/RARα is the formation of stable complexes with the HD-NCR (histone deacetylase recruiting nuclear co-repressor complex). Both fusion proteins bind to the members of the HD-NCR, N-CoR and SMRT through the CoR box region of RARα. This binding is ligand-dependent, as shown by the release after treatment with pharmacological doses of ATRA. In contrast to PML/RARα, PLZF/RARα contains a second binding site for the members of the HD-NCR in the PLZF portion of the fusion protein. This binding is ATRA-resistant and is mainly located in the POZ domain [6], [12]. The BTB/POZ domain has been shown to bind to SMRT, mSin3A and HDAC-1, all mediators of the transcriptional repression, in a ligand-independent manner [6]. The aberrant recruitment of HDAC activity deregulates the transcription of ATRA-induced genes, which are responsible for the differentiation of hematopoietic precursor cells. The critical residues for both the interaction with the HD-NCR as well as the capacity to form HMW complexes were exactly mapped to the BTB-POZ domain [11]. Furthermore, we have shown that the BTB/POZ domain mediates the oligomerization of PLZF/RARα. Point mutations in the BTB/POZ, which interfere either with the correct folding or with the charge of the pocket formed between the two POZ subunits, are able to inhibit the function of both PLZF and PLZF/RARα. These point mutations, which inhibit oligomerization, abolish the PLZF/RARα-related differentiation block and the aberrant self-renewal of PLZF/RARα-positive hematopoietic stem cells (HSCs) [13].

In this work, we tested whether we could target the oligomerization domain of PML/RARα or PLZF/RARα with interfering polypeptides that represent the respective oligomerization regions in PML and PLZF (PCC and POZ, respectively). We showed that the co-expression of the interfering peptides overcame the differentiation block induced by PML/RARα and PLZF/RARα (X-RARα, where X corresponds either to PML or PLZF) in murine hematopoietic stem cells, resulting in a reduced replating efficiency of the X-RARα-expressing HSCs in the same system. The peptides were able to bind to the fusion protein and modify the HMW complex formed by each X-RARα. In conclusion, specific molecular targeting by interfering with the oligomerization of the APL fusion proteins demonstrates a proof of principle for a promising therapeutic approach and represents an effective tool to further understand the biology of leukemia.

## Design and Methods

### Plasmids

The methods for the cDNAs encoding PCC, POZ and GFP were previously described [14]. Megaprimers were designed for the amplification and further subcloning of PCC/POZ-GFP using PCR. Megaprimer 1 contained the complete PCC/POZ and flanking GFP sequences with the primers [AAATGGAAGCTTCCACCATGACGCAGGCGCTGCAG-GAGC], [AAATGGAAGCTTCCACCATGGATCTGACAAAAATGGGC] and [AATACCAAGCTTTT-ACTTGTACAGCTCGTCCATGCC]. Megaprimer 2 contained the complete GFP and flanking PCC/POZ sequences with the primers [TTCGACGAGTTCAAGGTGCGCCTAATGGTGAGCAAGG-GCGAGGAGC], [ACAGCTCCTCGCCCTTGCTCACCATTAGGCGCACCTTGAACTCGTCG], [TGAAGATGCTGGAGACCATCCAGATGGTGAGCAAGGGCGAGGAGC] and [ACAGCTCCTCGC-CCTTGCTCACCATCTGGATGGTCTCCAGCATCTTCAGGC]. These megaprimers were further used to synthesize PCC/POZ-GFP using PCR. These sequences were inserted into the PIDE vector by digestion with the HindIII restriction enzyme.

The pE-PCC-ABL, pE-POZ-ABL, pE-GFP, pE-PML/RARα and pE-PLZF/RARα vectors for Gateway insertion of the fusion protein were described previously [13], [15]. For further subcloning of the cDNAs into different expression vectors, the Gateway recombination system (Invitrogen, Munich, Germany) was used. All cDNA sequences were cloned in the pENTR1A vector and shuttled with the LRClonase-Enzyme Kit (Invitrogen, Munich, Germany) into plasmids previously converted to Gateway destination vectors according to the manufacturer's instructions. For the immunoprecipitation experiments, we used a pCDNA3-derived vector to transiently express PML/RARα, PML/RARα or the peptides HA-PCC, HA-POZ, HA-PCC or HA-empty under the control of a CMV promoter. For retroviral transduction, we used the PINCO retroviral vector [16] and its derivative PIDE to generate PCC/POZ-GFP. The transgene expression was driven by the LTR and the expression of the enhanced green fluorescence protein (eGFP) [17] or the GFP fusion protein, under the control of the CMV promoter. PML3-160/RARα, in which the three SUMO-binding sites were mutated was graciously provided by Thomas Sternsdorf.

### Cell lines, cell culture and western blotting

Phoenix ecotropic packaging cells (Orbigen, San Diego, USA) were maintained in DMEM supplemented with 10% FCS (Invitrogen, Munich, Germany), 1% penicillin/streptomycin and 1% L-glutamine (Gibco-BRL, Invitrogen, Munich, Germany). BA/F3 (ACC-300 DSMZ) cells were maintained in RPMI supplemented with 10% FCS, 1% penicillin/streptomycin, 1% L-glutamine and mIL-3 (20 ng/ml) (Invitrogen, Munich, Germany).

Cell lysates were prepared in an SDS lysis buffer (1.5 M Tris-HCl, pH 6.8, 20% SDS, 10% Glycerol). The cells were treated for 16 h with 10 µM MG132 and 20 µM calpain I or for 24 h with 500 nM lactacystin.

Western blot analysis was performed using the anti-RARα (C-20 - Santa Cruz Biotechnology, Santa Cruz, USA), anti-GFP (FL - St. Cruz), anti-c-ABL (24-11, St. Cruz Biotechnology, Santa Cruz, USA) and anti-phospho-ABL (Tyr245, Millipore, Billerica, USA), anti-GAPDH (FL-335 - St. Cruz), and anti-tubulin (Ab-4, NeoMarkers, Thermo Scientific Inc., Kalamazoo, USA) antibodies. The membranes were blocked in 5% low-fat milk and washed in TBS/0.1% Tween20 (TBS-T). The antibodies were diluted in either 5% low-fat dry milk (anti-GFP, anti-tubulin), 0,5% low-fat dry milk (anti-GAPDH, anti-RARα) or TBS-T (anti-phospho-ABL, anti-c-ABL, anti-HA). Horseradish peroxidase-conjugated secondary antibodies (St. Cruz Biotechnology, Santa Cruz, USA) were diluted 1∶2000 in 5% low-fat dry milk or TBS-T.

### IL3 starvation assay in BA/F3 cells

BA/F3 cells were infected with retrovirus, as previously described.[14] The cells were cultured without IL3 for five days. Each day, the cells were counted, and the GFP content was measured by FACS.

### Size exclusion chromatography

Proteins were translated using the TNT®T7 Quick Coupled Transcription/Translation System (Promega, Madison, USA) according to the manufacturer's instructions. The protein solution was diluted with the chromatography running buffer.

Phoenix cells were transiently transfected using the calcium phosphate transfection method, as previously described [4]. After 3 days, the cells were incubated with 10 µM MG132 for 6 h. The cells were lysed with IPH lysis buffer (100 mM Tris, pH 8.0, 150 mM NaCl, 5 mM EDTA, 5% NP40) supplemented with Protease Inhibitor Cocktail Set I (Merck KGaA, Darmstadt, Germany) and Phosphatase Inhibitor Cocktail Set II (Merck KGaA, Darmstadt, Germany) and sonified (Amplitude: 20%, pulse: 1 sec, pause: 2 sec, duration: 30 sec) by a Digital Sonifier W-250D (Branson Ultrasonic Cooperation, Danbury, USA). Liquid chromatography was performed at 4°C using a Superdex 200 10/300 GL size exclusion column (GE-Healthcare, Upsala, Sweden) with a running buffer (20 mM Tris, pH 8.0, 175 mM NaCl, and 5% glycerol) at a flow rate of 0.4 ml/min. Half-milliliter fractions were collected, concentrated using StrataClean Resin (Agilent Technologies, Santa Clara, USA) and analyzed by western blot. Protein bands were then detected using the VersaDoc Imaging System (Bio-Rad, Munich, Germany), and a densitometric analysis was performed using Quantity One software (Bio-Rad, Munich, Germany).

### Immunoprecipitation

Using the calcium phosphate transfection method, the 293 cells were transiently transfected with the PCDNA3 vector expressing PML/RARα or PLZF/RARα (X-RARα, where X corresponded to either PML or PLZF) combined with HA-PCC, HA-POZ or HA-BCC constructs, as previously described [4]. Empty vectors or a vector containing only the HA tag were used as controls. The 293 cells were then lysed in buffer containing 100 mM NaCl, 50 mM Tris-HCl, 50 mM HEPES, 1 mM EDTA, 100 mM sodium pyruvate, 0.1% NP-40, 5 mM sodium orthovanadate and protease inhibitors (Roche Complete, Basel, Switzerland). Lysates were precleared for 1 h at 4°C on Protein-G Sepharose (GE-Healthcare, Upsala, Sweden). An anti-hemagglutinin (anti-HA) protein affinity matrix composed of rat monoclonal anti-HA antibody (clone 3F10) covalently coupled to agarose beads (Roche, Basel, Switzerland) was used to precipitate the HA-tagged peptides. The beads were subsequently washed in the buffer described above and re-suspended in LDS loading buffer containing a reducing agent; 10% of the input served as a control. The samples were analyzed by western blot and stained with an anti-HA antibody to detect the peptides and with anti-RARα antibodies to detect X-RARα.

### Isolation of murine Sca1^+^/lin^−^ hematopoietic progenitor cells (m-HPCs), retroviral infection, colony assay and differentiation analysis

The isolation and retroviral infection of the m-HPCs were performed as previously described [15]. The infection was repeated in 3 alternating rounds for each construct. Infected cells were assessed by GFP detection.

The m-HPCs [15] were cultivated in DMEM supplemented with 1% penicillin/streptomycin, 1% L-glutamine (Gibco-BRL, Invitrogen, Munich, Germany) 10% FCS, mIL-3 (20 ng/mL), mIL-6 (20 ng/mL) and mSCF (100 ng/mL) (StemCell Technologies, Vancouver, Canada). On day 3, the cells were plated in triplicate at a concentration of 5000 cells/ml in semisolid methylcellulose without EPO (Stem Cell Technologies, Vancouver, BC Canada), supplemented with cytokines from StemCell Technology and incubated at 37°C with 5% CO_2_. After a 10-day incubation, the cells were counted and analyzed for differentiation by morphological analysis and FACS. The cells were washed, and the surface markers Sca1, cKIT, Gr-1 and Mac-1 were assessed with PE-conjugated antibodies (Becton Dickinson, Heidelberg, Germany) and detected by FACS. A total of 100,000 cells were cytofuged and stained with a Wright-Giemsa solution to morphologically analyze their differentiation state.

### Reverse Transcriptase-PCR (RT-PCR)

Total RNA and first-strand cDNA were obtained from the m-HPCs (3 days after infection) according to standard protocols. PCR was conducted following standard protocols using a Robocycler (Perkin Elmer Cetus, Norwalk USA). The PCR products were analyzed by agarose gel electrophoresis. The quality of the cDNA was assayed by a β-actin PCR amplification. For the specific amplification of the breakpoints following the primer, the following were used: for PML/RARα, PML-A1, 5′-CAGTGTACGCCTTCTCCATCA-3′ and RARα-B, 5′-GCTTGTAGATGCGGGGTAGA-3′ [18]; for PLZF/RARα, PLZF-24, 5′-GCTGACGCTGTATTGAGC-3′ and RARα-24, 5′-ACATGCCCACTTCAAAGC-3′.

## Results

### Targeting PML/RARα and PLZF/RARα through peptide interference

We previously showed that the PLZF/RARα oligomerization is responsible for repressing promoter activity [13] and biological function, as indicated by the inability of PLZF/RARα to induce the differentiation block and the aberrant stem cell capacity through the expression of oligomerization defective PLZF/RARα mutants.

To target the oligomerization domains of PML/RARα and PLZF/RARα, we used a peptide that represents the functionally “coiled-coil” oligomerization surface of PML/RARα (PCC; aa. 221–361) or the POZ domain of PLZF (aa. 1–125), which represents the oligomerization surface of PLZF/RARα.

As shown previously, the forced oligomerization of ABL kinase causes IL3-independent growth in Ba/F3 cells [14]. To investigate whether the peptides were able to inhibit the biological function of the fusion proteins, we exploited the ability of the oligomerization interfaces fused to ABL to cause growth factor-independent Ba/F3 cell growth. We generated constructs to co-express the fusion protein and the respective peptide. We subcloned the PCC and the POZ peptides fused to GFP into retroviral vectors expressing PCC/ABL and POZ/ABL fusion proteins, respectively.

**Figure 1:**
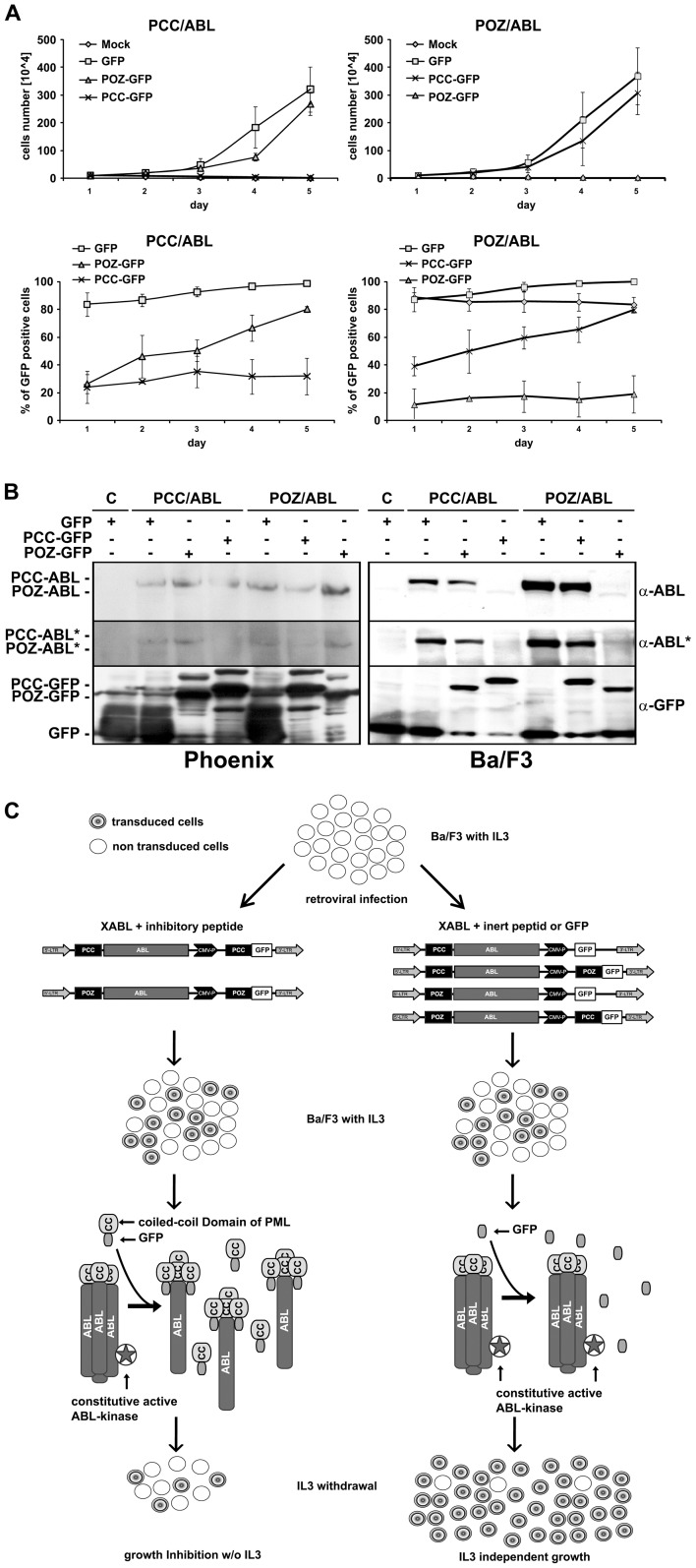
Co-expression of PCC/POZ-GFP reverses IL3-independent growth in PCC/ABL- or POZ/ABL-positive BA/F3 cells. *A*, Infected BA/F3 cells were cultivated for 5 days without IL3. The total cell count and FACS measurement of the GFP signal were ascertained daily, n = 3. The upper panels represent the GFP positive population accumulation, and the lower panels represent the IL3-independent growth of the Ba/F3 cells expressing the ABL fusions in the presence or absence of the related peptides or GFP alone, as indicated. Mock: PIDE vector without BCC-ABL or POZ-ABL, expressing only GFP. *B*, Western blot of whole cell lysates of Phoenix and BA/F3 cells probed for ABL (α-ABL), phospho-ABL (α-ABL*) and GFP (α-GFP). Control: empty vector. *C*, Schematic diagram of IL3 growth dependency, as shown for PCC. By fusing ABL to PCC, BA/F3 cells can grow in the absence of IL3. The introduction of PCC-GFP inhibits the PCC/ABL complex formation and restores IL3-dependent growth.

To assess the functionality, these constructs were transduced into Ba/F3 cells, and the derived cells were tested for IL3-independent growth (Figure 1C).

As expected, the Ba/F3 cells expressing PCC/ABL or POZ/ABL grew in the absence of IL3, whereas cells carrying empty retrovirus alone (Mock) did not. In contrast, the cells co-expressing PCC-GFP or POZ-GFP (also called interfering peptides) showed inhibited cell growth in the absence of IL3 (Figure 1A).

As controls, we used GFP alone or the interfering peptide of the other fusion protein. In this way, we were able to show that the growth inhibition was specifically due to the co-expression of the peptide that targeted the oligomerization. The growth of the Ba/F3 cells expressing PCC-ABL was suppressed with the PCC-GFP peptide, but not with GFP alone or the POZ-GFP. Similar results were observed with the POZ/ABL construct and the respective controls (Figure 1 A, C). In these experiments, the infection efficiency was different for each viral vector. We repeated the experiments with the cells expressing the same amount of GFP at the starting point. As shown in Figure S1, we observed similar results to those previously obtained. To assess the expression of the proteins, we performed western blotting with the cell lysates of these cells. PCC-ABL and POZ-ABL were expressed in the retrovirus-producing Phoenix cells (Figure 1B). The phosphorylated ABL protein was also detected in these cells, appearing less phosphorylated in the presence of the corresponding inhibitory peptide (Figure 1B). In the infected Ba/F3 cells, there was no or little expression of these proteins in the presence of the corresponding interfering peptide. Only a small amount of the protein was detected with the anti-phospho-ABL antibody in the Ba/F3 cells. These data show that the proteins were correctly expressed in the Phoenix cells. In contrast, the expression was not detectable in Ba/F3 cells, most likely due to a cell type-dependent protein degradation, as discussed below.

### The interfering peptides bind to the X-RARα fusion protein

**Figure 2:**
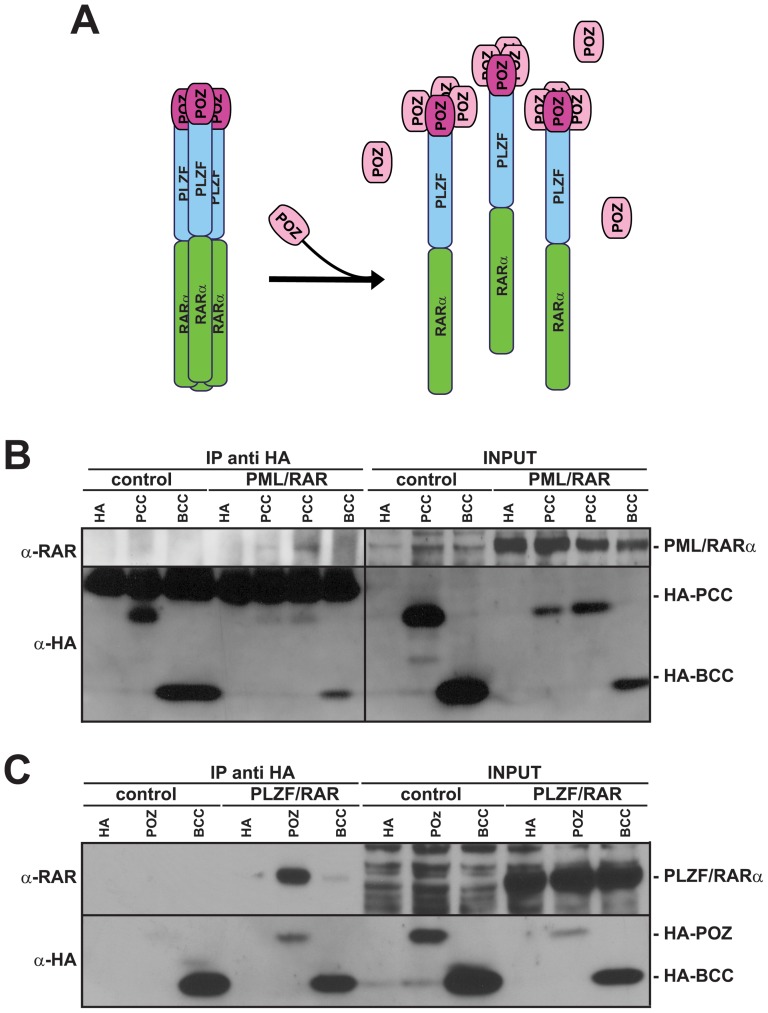
PCC and POZ bind to PML/RARα and PLZF/RARα, respectively. *A*, Theory for the peptide binding to X-RARα, as exemplified by POZ and PLZF/RARα. By binding the oligomerization domain of PLZF/RARα, POZ disables the self-oligomerization and the high-molecular-weight complex formation. *B*, *C*, HA-tagged peptides were co-expressed with PML/RARα or PLZF/RARα in the 293 cells through the transfection of the corresponding combination of pCDNA3 vectors with calcium phosphate. An HA-empty plasmid and an HA-tagged BCC (coiled-coil domain of BCR) were used as a specificity control. In *B*, PCC was transfected at two different concentrations, 5 and 10 µG DNA (first and second BCC lane, respectively). Immunoprecipitation (IP) was performed with an anti-HA matrix. Western blots were probed with α-HA and α-RARα antibodies.

The PCC or POZ domains are responsible for the homomeric binding of PML/RARα and PLZF/RARα, respectively [7], [13], [19]. Our interfering peptides, which bind to these domains, should be able to complex with X-RARα (Figure 2A). To test this hypothesis, we co-expressed HA-tagged PCC/POZ and X-RARα in the 293 cells and performed co-immunoprecipitation experiments. As expected, both fusion proteins co-immunoprecipitated with the corresponding interfering peptide (Figure 2B). For PML/RARα and PCC, the binding appeared to be stoichiometric, as shown by the use of different amounts of PCC. Another unrelated “coiled-coil” domain (BCC; from BCR) and HA alone demonstrated no binding to PML-RARα. Although the BCC lane was dark, this darkness was due to the background, as no specific band was visible. This result shows that the interaction is specific (Figure 2B). Strong and specific binding was shown for PLZF/RARα and POZ (Figure 2C). In conclusion, these data show that each peptide is able to bind to the related fusion protein.

### Effect of the interfering peptides on the high-molecular-weight complexes formed by the X-RARα fusion protein

**Figure 3:**
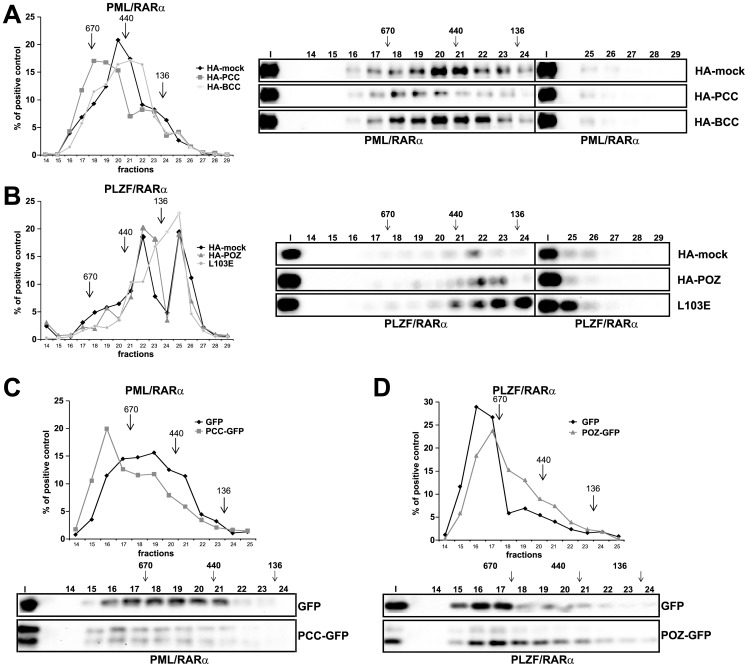
PCC and POZ influences on the HMW complex formation of PML/RARα and PLZF/RARα, respectively. *A and B*, Size-exclusion chromatography fractions of *in vitro* translated PML/RARα (*A*) or PLZF/RARα (*B*) and HA-tagged peptide. Fractions were analyzed by western blot (on the right) and probed against RARα. The densitometric analysis of the western blot is shown on the left side as a percentage of the entire signal. *C and D*, Size-exclusion chromatography of the Phoenix whole cell lysates overexpressing PML/RARα (*C*) or PLZF/RARα (*D*) and GFP-tagged peptide. Fractions were analyzed by western blot (WB) and probed against RARα (α-RARα) (lower part). The densitometric analysis of the western blot is shown in the upper part as a percentage of the entire signal. HA-mock: control empty vector. Input (I), eluted fractions 14 to 29. The numbers above the arrows represent the molecular weight (KD) of the proteins used for the MW calibration: 136 KD for the BSA dimer, 440 KD for Ferritin, and 670 KD for Thyroglobulin.

Previously, we showed that the PLZF/RARα complex formation is essential for the incorrect gene regulation caused by PLZF/RARα, resulting in a differentiation block [13]. We tested the ability of X-RARα to form complexes in the presence of the interfering peptide. For the *in vitro* analysis, we translated X-RARα together with either the related peptide or a mock control and analyzed the protein size using size exclusion chromatography. As shown in Figure 3, the *in vitro* translated PML/RARα in the presence of the HA peptide alone formed a complex with a maximum peak eluting between 381 and 484 KD. The presence of the HA-PCC peptide increased the complex size to 670 KD. This increase was not observed with the BCC domain. For PLZF/RARα, the *in vitro* translated protein formed a complex that eluted with a maximum peak at fraction 22, with a molecular weight of 236–300 KD. The elution peak was only partially reduced to fraction 23 (185–236 KD) in the presence of the HA-POZ peptide. Fractions 17 to 20 of PLZF/RARα had a higher degree of intensity compared with those fractions eluting in the presence of the HA-POZ peptide. As a control, we used a POZ mutant of PLZF/RARα, PLZFL103E/RARα. As reported previously [13], this mutant lost the ability to form HMW complexes and eluted at fraction 25 with a molecular weight less than 136 KD, which most likely represented the monomeric form of the protein (Figure 3B).

We proceeded to validate these results in the intracellular context. We co-expressed X-RARα with the related interfering peptide in Phoenix cells. After lysis under non-denaturing conditions, the protein extracts were subjected to a chromatographic analysis. Under these conditions, the PML/RARα fusion protein formed a complex that eluted in fractions 17 to 21, similar to the results previously observed [7], [20]. In the presence of PCC, the complexes eluted with a high peak at fraction 16 (Figure 3C), confirming the results obtained by the *in vitro* studies, but with an overall higher molecular weight. For PLZF/RARα, the complex eluted in fraction 16. In the presence of the HA-POZ peptide, this protein was destabilized, eluting in the lower molecular weight fractions.

In summary, these data show that the two X-RARα complexes were modified by the presence of the peptide in different ways: the PML/RARα complex became augmented, whereas the PLZF/RARα complex was destabilized.

### The interfering peptides target the X-RARα fusion protein through protein degradation

To further investigate the effects of the interfering peptides on the leukemic phenotype, viral supernatant from the Phoenix cell line was used to infect Sca1^+^/lin^−^ murine hematopoietic progenitor cells (m-HPCs) with the indicated constructs.

**Figure 4:**
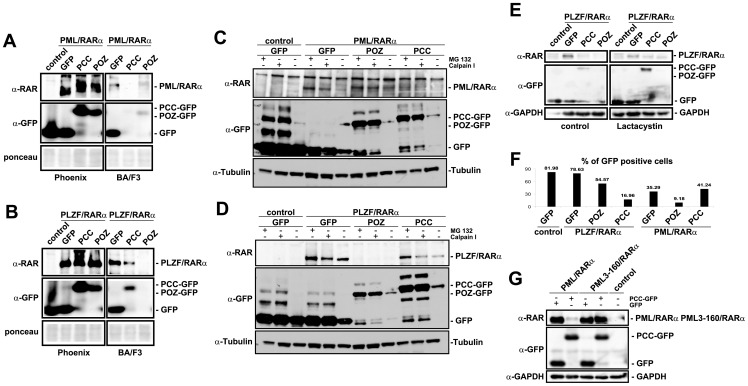
PM/RARα and PLZF/RARα, degrade in the presence of PCC and POZ, respectively. *A and B*, Western blot of the whole cell lysates of PML/RARα- (*A*) or PLZF/RARα (*B*)-positive Phoenix and BA/F3 cells probed against RARα (α-RARα) and GFP (α-GFP). *C and D*, BA/F3 cells were treated with 10 µM MG132 or 20 µM Calpain I for 16 h. Control: empty vector; tubulin: loading control. *E*. BA/F3 cells were treated with 500 nM Lactacystin for 24 h. Control: empty vector; GAPDH: loading control. *F*, The infection efficiency, measured as the percentage of GFP-positive cells, of the Ba/F3 cells after infection with PINCO (control) or PIDE carrying PLZF/RARα alone or in combination with GFP, GFP-POZ or GFP-PCC or PML/RARα alone or in combination with GFP, GFP-POZ or GFP-PCC peptides, as indicated. *G*, Western blot of the Phoenix whole cell lysate expressing PML/RARα or a sumoylation-deficient mutant (PML3-160/RARα) PCC-GFP or GFP, probed with α-GFP, α-RARα and α-GPADH as a loading control. The image shown is a representative of three separate experiments.

The infection efficiency of the retroviral supernatant in Ba/F3 cells was measured. The supernatant was then used to infect the m-HPCs. Infected Ba/F3 cells were lysed and analyzed by western blot to confirm the expression of the expected proteins. We observed that PML/RARα and PLZF/RARα were not detectable in the samples when the interfering peptide was co-expressed (Figure 4 A–B).

To investigate if the PML/RARα and PLZF/RARα fusion proteins were not detected in the presence of the interfering peptides due to a proteasomal degradation, we treated the cells with proteasome inhibitors. The infection efficiency of these cells was measured with FACS as the percentage of GFP-positive cells (Figure 4F). We observed that the PML/RARα protein was stabilized by the presence of MG123 and the calpain I inhibitor but the protein degradation was not completely impaired (Figure 4C). In addition, the PML/RARα protein co-expressed with GFP alone or POZ-GFP appeared to be stabilized in the presence of the inhibitors (Figure 4C). The degradation of PLZF/RARα in the presence of the POZ-GFP peptide appeared to be not inhibited by MG 132 or Calpain I (Figure 4D). A more specific and irreversible proteasome inhibitor for PLZF/RARα, Lactacystin, appears to at least partially restore the expression of PLZF/RARα in the presence of POZ-GFP (Figure 4E). These data strongly suggest that the interfering peptides partially trigger the proteasomal degradation of the PML/RARα and the PLZF/RARα fusion proteins.

### SUMO modifications of PML-RARα are indispensable for the PCC-GFP peptide-dependent degradation

PML/RARα is modified by PIC1/SUMO prior to degradation [21], [22]. To test whether this modification plays a role in the interfering peptide-induced degradation of PML/RARα, we used a mutant of PML/RARα (PML3-160/RARα) in which the three SUMO-binding sites were mutated. As shown in Figure 4 (panel G), we observed a strong reduction in the PML/RARα protein band intensity in the presence of the PCC-POZ peptide, while the PML3-160/RARα mutant showed no significant change in the presence of the peptide. In conclusion, we suggest that the SUMO modification is indispensable in the degradation of PML/RARα induced by the PCC peptide.

### The interfering peptides ameliorate the X-RARα-induced myeloid differentiation block in murine hematopoietic stem cells

The ability of the interfering peptide to influence the differentiation block and the aberrant self-renewal induced by X-RARα was investigated.

**Figure 5:**
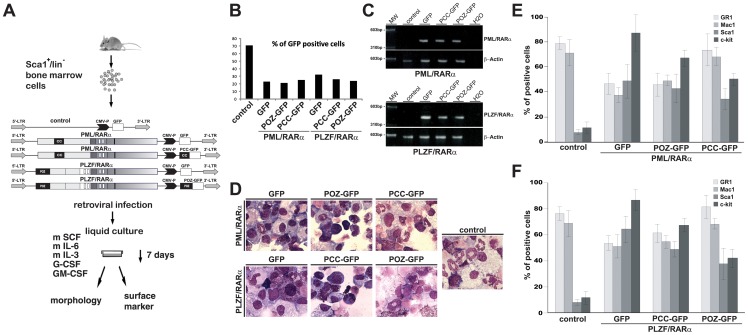
PCC and POZ reverse the differentiation block of PML/RARα- and PLZF/RARα-positive murine hematopoietic stem cells. *A*, Schematic diagram of the experiment conditions. Sca1^+^/lin^-^ murine bone marrow cells were isolated and infected with retroviral vectors containing PML/RARα or PLZF/RARα and GFP or PCC/POZ-GFP and then analyzed for differentiation. *B*, The infection efficiency, measured as the percentage of the GFP-positive cells to the retrovirally infected cells. *C*, Reverse transcriptase-PCR of the retrovirus-infected Sca^+^/lin^−^ bone marrow cells for PML/RARα and PLZF/RARα. Control: β-Actin. *D*, GIEMSA staining of Sca^+^/lin^−^ bone marrow cells seven days after infection with retroviral vectors. *E*, FACS analysis of the Sca1^+^/lin^−^ bone marrow cells infected with PML/RARα and GFP or PCC/POZ-GFP. Mock: empty vector. *F*, FACS analysis of the Sca1^+^/lin^−^ bone marrow cells infected with PLZF/RARα and GFP or PCC/POZ-GFP. Mock: empty vector.

X-RARα in the presence or the absence of the corresponding interfering peptide or an unrelated control peptide was expressed in early m-HPCs and the state of differentiation was measured. A diagram of the experimental strategy is shown in Figure 5A. The infection efficiency of the control cells harboring the PINCO empty (control cells) was 71%. For the fusion construct, the infection efficiency was between 21% and 32%, as shown in Figure 5B. To determine whether the fusion proteins were effectively expressed in the m-HPCs, RNA was extracted, and RT-PCR was performed using primers directed against the specific translocation break point (for PML/RARα PML-A1 and RARα-B [18]; for PLZF/RARα with the primers PLZF-24 and RARα-24; see methods section for sequences). As expected, we were able to detect the transcripts (Figure 5C). We found that X-RARα caused a differentiation block in the m-HSCs, which was reversed by the introduction of the interfering peptide, as represented as the larger and differentiated cells shown in Figure 5D. The co-expression of the interfering peptide led to an increased expression of Gr-1 and Mac-1 surface markers to levels comparable to the control cells, indicating an induced differentiation (Figure 5 E–F). A reduction in the expression of Sca1 and c-Kit indicated a reduction in the stem cell compartment (Figure 5 E–F). This effect was specific because the unrelated control peptides did not show a similar effect.

In summary, the PML/RARα- and PLZF/RARα-related differentiation block in the m-HPCs was reverted by co-expressing PCC-GFP or POZ-GFP, respectively.

### The interfering peptides abrogate the X-RARα-aberrant replating efficiency in murine hematopoietic stem cells

**Figure 6:**
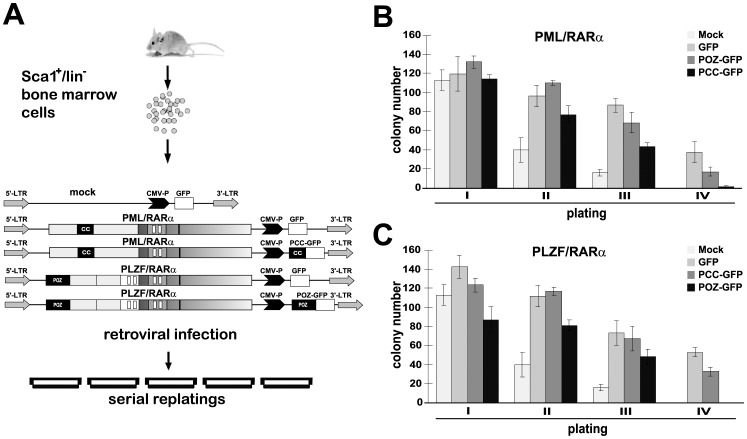
PCC and POZ reverse the proliferation capacity of PML/RARα- and PLZF/RARα-positive murine hematopoietic stem cells. *A*, Schematic diagram of the experimental conditions. Sca1^+^/lin^−^ bone marrow cells were isolated and infected with retroviral vectors containing PML/RARα or PLZF/RARα and GFP or PCC/POZ-GFP. Infected cells were plated in methylcellulose on day 3. Colony counts and replating were measured every 10 days. *B* and *C*, Colony count of Sca1^+^/lin^−^ bone marrow cells infected with PML/RARα (*B*) or PLZF/RARα (*C*) and GFP or PCC/POZ-GFP cultured in methylcellulose. Mock: empty vector.

To investigate if the interfering peptides were able to abrogate the aberrant replating efficiency of PML/RARα- and PLZF/RARα-expressing m-HPCs, we performed serial replating experiments with the infected m-HPCs expressing PML/RARα or PLZF/RARα in the absence or presence of the respective interfering GFP peptides, as discussed above (Figure 5A). The PCC-GFP peptide diminished the colony numbers in each passage, abrogated the capacity of PML/RARα-expressing cells to be replated more than 4 passages (Figure 6B) and restored the normal phenotype. The same effect was observed with PLZF/RARα. The POZ-GFP co-expression strongly inhibited the colony numbers at passages 2 and 3 and did not permit replating more than 4 times (Figure 6C).

These data show that the interfering peptides can effectively target the X-RARα fusion proteins and abolish their serial replating capacity.

## Discussion

In this work, we targeted APL-related fusion proteins using interfering peptides directed specifically against the oligomerization domain.

To assess this approach in a biological system, the ability of the protein kinase ABL to become activated if fused to an oligomerization surface [14] was determined and used as a proof of principle. We used an artificial system that fused the “coiled-coil” of PML or the BTB/POZ domain of PLZF to ABL. These fusion proteins were previously reported to induce growth factor-independent Ba/F3 growth [14].

With this approach, we showed that the co-expression of the interfering peptides targeted against the oligomerization domain abolishes the capability of the fusion protein to induce the growth factor-independent phenotype in Ba/F3 cells.

This approach represents an effective and easy method that can be reproduced with different oligomerization surfaces to test the ability of peptides and drugs to interfere with protein oligomerization.

The biological activity of the PML/RARα and PLZF/RARα fusion proteins depends on the presence and the integrity of their oligomerization domains [9], [13], [23].

We first tested the ability of the interfering peptides to bind to their respective fusion protein by co-immunoprecipitation experiments using HA-tagged peptides and found that the peptides specifically bound to their respective fusion proteins. We performed several immunofluorescence experiments, but we failed to see a co-localization between the fusion proteins and the peptides, probably due to the fact that the expression of the fusion protein is so strong repressed by the co-expression of the interfering peptide. We believe that the co-immunoprecipitation indicates that the peptide bound to the corresponding protein in our system. Multiple reports have already demonstrated the binding to the oligomerization surface ([7] [24] [20] [10] [13]. We then tested the effect of the peptides on the fusion protein complex formation. We showed that the PCC does not destroy the HMW complex formation but rather generates complexes with a higher molecular weight in combination with PML/RARα. This finding was surprising, but in accordance with other mechanisms involved in the PML/RARα inactivation, such as the effect of arsenic trioxide. Arsenic trioxide is able to directly bind to the N-terminal region of PML and induce an enhanced oligomerization, followed by a subsequent degradation upon the recruitment of SUMO [25].

The forced expression of an isolated “coiled-coil” domain can be assumed to bind with higher intensity to the wild type, inducing a SUMO-dependent proteasomal degradation. The binding of PCC and SUMO to PML/RARα may have caused the augmented complex sizes that were observed in our experiments. In this work, we showed that the SUMO modification of PML/RARα is indispensable for protein degradation, as indicated by the failed degradation of the SUMO-mutated PML/RARα in the presence of the interfering peptide.

The co-expression of PLZF/RARα and the POZ-GFP peptide interfered with the formation of the HMW complexes, reducing their size to a lower molecular weight. This observation is consistent with a previous report on the AML1/ETO HMW complex inhibition by an interfering peptide [26].

We also tested the effect of the peptide on the biology of X-RARα. PML/RARα and PLZF/RARα are known to induce specific phenotypes if expressed in Sca1+/Lin− hematopoietic murine progenitor cells. In m-HPCs, X-RARα blocks terminal differentiation and causes abnormal self-renewal. In this work, we investigated the ability of the interfering peptides to inhibit the leukemic phenotype induced by X-RARα and found that the co-expression of these peptides with their respective X-RARα overcomes the X-RARα-induced differentiation block and self-renewal. A similar effect was shown by interfering with the oligomerization of the AML1/ETO fusion protein using a peptide designed to bind to the oligomerization domain [26]. Targeting the fusion protein/co-repressor contact was reported to restore a differentiation response in leukemia cells expressing PML/RARα or AML1/ETO [27].

The expression of interfering peptides specifically induced the degradation of the PML/RARα and PLZF/RARα protein. A similar result was previously described for interfering peptides designed to bind to the fusion protein/co-repressor contact region of PML/RARα [27]. The proteasomal inhibitors abolished the degradation, suggesting that the fusion protein is degraded by the proteasome. The use of MG123 or Lactacystin for PLZF/RARα only partially restored the fusion protein expression, suggesting that this degradation is only mediated in part by the proteasome. One possible mechanism might be that the “coiled-coil” domain of PML is a target for the E3 Ubiquitin-Ligase SIAH1/2, which mediates the degradation of PML/RARα [28]. In our study, we showed that the complex formed by PML/RARα increases in size, suggesting that the addition of PCC peptides triggers a degradation that is dependent upon a binding to PIC/SUMO1.

In the first part of this work, we showed a promising model for screening small peptides or low-molecular-weight compounds designed to block oligomerization using oligomerization domains fused to the ABL kinase. This approach could provide a significant improvement for the therapeutic treatment of acute myeloid leukemia and other diseases in which protein oligomerization plays a dominant role.

We showed that the X-RARα oligomerization domain is a promising target for molecular intervention. The oligomerization domain in the fusion protein was a key component contributing to the fusion protein's oncogenic properties. The targeting of the oligomerization led to an inhibition of the leukemic phenotype and was accompanied by a strong protein degradation that efficiently destroyed the oncogenicity of the fusion protein.

## Supporting Information

Figure S1 **Co-expression of PCC/POZ-GFP reverses IL3-independent growth in PCC/ABL- or POZ/ABL-positive BA/F3 cells.** *A–D* Infected BA/F3 cells were cultivated for 6 days without IL3. The total cell count and FACS measurement of the GFP signal were ascertained daily, n = 2. The A–B panels represent the GFP positive population accumulation, and the C–D panels represent the IL3-independent growth of the Ba/F3 cells expressing the ABL fusions in the presence or absence of the related peptides or GFP alone, as indicated. *E–F*, Western blot of the whole cell lysates of the Phoenix and BA/F3 cells probed for ABL (α-ABL) and GFP (α-GFP). Control: empty vector.
(PDF)
