# Supplementary figures and images for: Targeting the Acute Promyelocytic Leukemia-Associated Fusion Proteins PML/RARα and PLZF/RARα with Interfering Peptides
Source: PLoS One. 2012 Nov 9;7(11):e48636. doi: 10.1371/journal.pone.0048636 (PMC3494703; doi:10.1371/journal.pone.0048636)

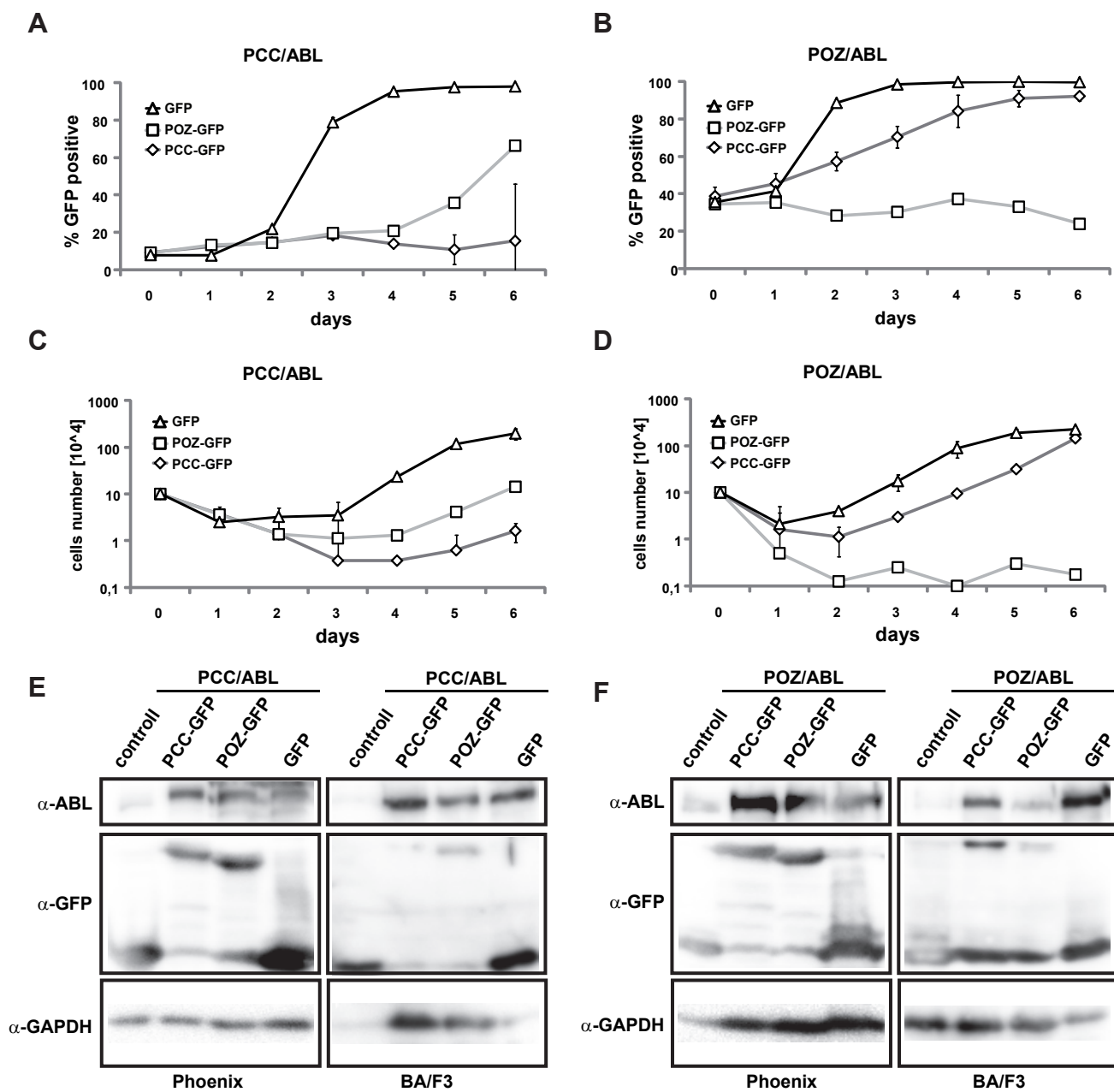

Beez Supplementary Figure 1

Supplement: Figure S1 — Co-expression of PCC/POZ-GFP reverses IL3-independent growth in PCC/ABL- or POZ/ABL-positive BA/F3 cells. A–D Infected BA/F3 cells were cultivated for 6 days without IL3. The total cell count and FACS measurement of the GFP signal were ascertained daily, n = 2. The A–B panels represent the GFP positive population accumulation, and the C–D panels represent the IL3-independent growth of the Ba/F3 cells expressing the ABL fusions in the presence or absence of the related peptides or GFP alone, as indicated. E–F, Western blot of the whole cell lysates of the Phoenix and BA/F3 cells probed for ABL (α-ABL) and GFP (α-GFP). Control: empty vector. (PDF) [file pone.0048636.s001.pdf]
